# Unexpected bonds: ubiquitin-like conjugation of cGAS/CD-NTases supports their enzymatic activity and antiphage defense

**DOI:** 10.1038/s41392-023-01549-7

**Published:** 2023-08-25

**Authors:** Katarzyna Andryka-Cegielski, Sofía Soler, Eva Bartok

**Affiliations:** 1 Institute of Experimental Haematology and Transfusion Medicine, University Hospital, University of Bonn, Bonn, Germany; 2 Institute of Clinical Chemistry and Clinical Pharmacology, University Hospital, University of Bonn, Bonn, Germany

**Keywords:** Innate immunity, Microbiology, Infectious diseases

In their recently published study in *Nature*, Jenson et al.^1^ provide compelling, unexpected evidence that bacterial cGAMP synthase (cGAS) and other cGAS/DncV-like Nucleotidyltransferases (CD-NTase) undergo ubiquitin-like conjugation (ULC) in type-II cyclic-oligonucleotide-based anti-phage signaling systems (CBASS). cGAS conjugation promotes cyclic-nucleotide (CN) formation and thus antiphage defense. Furthermore, the authors also reveal a new class of phage-encoded CBASS inhibitor, T4 Vs.4, a CN-sponge which limits CBASS effector function and promotes phage replication, revealing another weapon in the “arms race” between phages and their bacterial hosts.

CN-based signaling is a common element in the antiviral immunity of bacteria, archaea, and metazoans. Bacterial CBASS is essential to antiphage defense and is closely evolutionarily linked to the metazoan antiviral pathway cGAS /STimulator of INterferon Genes (STING). In vertebrates, cGAS has evolved into a receptor for double-stranded DNA, which, upon activation, catalyzes the formation 2’-3’-cGAMP, the ligand for STING, leading to the production of the key vertebrate antiviral cytokine, type-I interferon. This pathway is essential to our own immune defense against numerous bacterial and viral pathogens.

As a minimal functional unit, CBASS contains two similar genes: (i) CD-NTase (also termed cGAS in *E. coli*), which, after an unknown activation mechanism, catalyzes the synthesis of CN second messengers and (ii) Cap, which, like metazoan STING, senses CN and carries out signaling and effector functions, including cell death programs. However, the type-II CBASS operon also encodes two ancillary genes, Cap2 and Cap3^2^ (Fig. 1), that surprisingly contain domains resembling eukaryotic ubiquitin-activating (E1, Cap2), -conjugating (E2, Cap2) and -deconjugating (JAB/JAMM, Cap3) enzymes. Little is known about protein conjugation in bacteria, and, although it had previously been shown that the enzymatic activities of Cap2 and Cap3 promote antiphage defense,^3^ the potential targets of these de/conjugating enzymes and their downstream molecular mechanisms remained completely unclear.

**Fig. 1:**
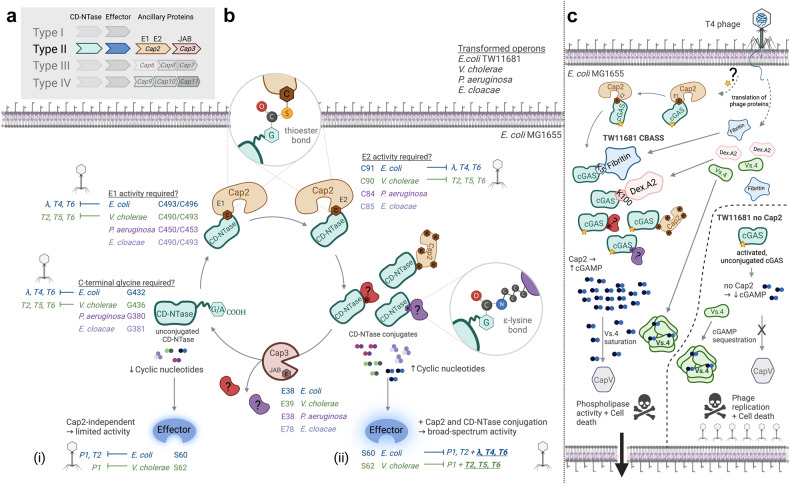
Ubiquitin-like conjugation of cGAS and other CD-NTases supports their enzymatic activity and antiphage defense. **a** An overview of known CBASS operons, type-II CBASS including Cap2 and Cap3 is highlighted. **b** The function of Cap2 and Cap3 and their contribution to cyclic-nucleotide (CN) signaling and anti-phage defense, according to Jenson et al.: Cap2 function increases CN production and broadens the repertoire of phages sensitive to type-II CBASS. Critical amino acids for the function of each protein in the operon are presented, color-coded for species. The TW11681 operon can defend against a limited repertoire of phages with (i) the effector protein but without ubiquitin-like conjugation when compared to (ii) a completely functional operon. **c** An overview of T4 phage infection in *E.coli* MG1655 transformed with the TW11681 operon. T4 phage infection leads to the production of the phage proteins Fibritin, Dex.A2 and Vs.4 (among others). If Cap2 is functional, Fibritin, Dex.81A2 and other targets are then cGASylated, leading to increased cGAMP production. Vs.4 acts as a cGAMP-sponge, raising the threshold for CapV-effector activation and phospholipase-mediated cell lysis. Without Cap2, cGAMP levels are not high enough to activate CapV-dependent cell death, permitting phage replication and also ultimately killing the bacterium. This figure was created using BioRender.com

To address these questions, Jenson and colleagues utilized a straightforward but effective approach: *E.coli* MG1655, which lacks CBASS, was used for the expression of diverse type-II CBASS systems^3^ and modifications thereof throughout the manuscript (Fig. 1). In initial experiments, FLAG-Cap2 was incorporated into *E. coli* TW11681 CBASS to identify interactors via immunoprecipitation, and putative loss-of-function (LOF) Cap2 and Cap3 mutants^3^ were generated, targeting the predicted active sites of E1 (C493A/C496A), E2 (C91A) and JAB (E38A). Here, retrieval of FLAG-Cap2 revealed a surprising target: *E. coli* cGAS, itself, in E1- and E2-dependent thioester linkage. This was particularly unexpected since cGAS lacks both the dual C-terminal glycines common to most Ubiquitin-like proteins (ULP) and the β-grasp domain found in all ULP to date.

Nonetheless, the study amasses substantial evidence to support this unexpected “cGASylation”. Inclusion of FLAG-cGAS in the TW11681 operon demonstrated extensive cGASylation of proteins; mass spectrometry later confirmed C-terminal glycine (CTG)-lysine/ε-amine-binding to targets, as is found in eukaryotic ubiquitination, and alignment of type-II CBASS CD-NTases revealed conservation of the CTG or C-terminal alanine (CTA). CTG deletion (ΔCTG) or radical amino-acid replacement abrogated cGAS-Cap2 thioester formation and protein conjugation, while, interestingly, G432A mutation decreased, but did not eliminate, total cGASylation while increasing overall cGAS-Cap2 linkages, indicating G/A-differences in protein conjugation affinity. The mutations ΔCTG and G432A are subsequently used as important tools throughout the study to determine the contribution of cGASylation to signaling and anti-phage defense.

Identifying cGAS as the ULP also enabled characterization of Cap3/JAB-function: recombinant Cap3 was shown to cleave cGAS isopeptide-linkages, while LOF-Cap3 (E38A) mutation increased total cGAS conjugation. Importantly, similar CD-NTase-conjugating activity and enhancement by LOF-Cap3 could also be observed with the type-II CBASS operons of *Vibrio cholerae*, *Pseudomonas aeruginosa*, and *Enterobacter cloacae*, demonstrating that "CD-NTase-ylation” is a common type-II CBASS feature. Although specific dependence on Cap2 activity and ΔCTG could only be shown for *P. aeruginosa*, due to weak signal-to-noise for *V. cholerae* and *E. cloacae*, a recent publication has also confirmed the contribution of Cap2 to CD-NTase conjugation for these two species.^4^

Logically, the authors then addressed whether “CD-NTase-ylation” contributed to antiphage defense and CN production. Again, they used mutated operons to directly compare CD-NTase-conjugation (ΔCTG) and mutagenesis (G432A) to LOF-variants of other CBASS proteins.^3^ These data demonstrated a broad functional equivalence between Cap2 function and cGAS/CD-NTase-ylation: titers increased by Cap2-LOF (*E. coli*: λ, particularly T4, T6; *V. cholerae*: T2, T5, T6) were similarly impacted by ΔCTG, while phages only requiring CD-NTase and Cap-effector function for their inhibition (*E. coli*: P1, T2; *V. cholerae*: P1) and those insensitive to CBASS (*E. coli*: T5, *V. cholerae*: λ, T4) remained unaffected by ΔCTG mutations. Moreover, anti-T4-phage defense in TW11681 CBASS was found to be completely dependent on cGASylation (ΔCTG), rendering it an excellent model (T4/TW11681) for subsequent experiments.

Since CD-NTase-conjugation enhanced type-II CBASS function, it was hypothesized to increase CN production. Using T4/TW11681, cGAMP production after phage infection was compared between operons with different CBASS-LOF mutations, including Cap2 mutants and ΔCTG, demonstrating that cGASylation indeed increased cGAMP production. The authors then used T4/TW11681 with FLAG-cGAS to retrieve cGASylation targets and identify them by mass spectrometry. Although the cGAS-sidechain could be found on the ε-lysines of protein targets from host and phage, unfortunately, no overarching pattern could be discerned. Although most targets had one, unique conjugation site, Cap2 had five (beyond its thioester bonds). Moreover, while cGASylation was found to target two T4-phage proteins, Fibritin and Dex.A2 (Fig. 1), it remains unclear how this affects anti-phage defense. The pattern and function of cGASylation of host and phage proteins will likely be addressed in future studies.

Finally, the T4/TW11681 model was used to screen for anti-CBASS factors using hydroxylamine mutagenesis, revealing Vs.4 as a key phage protein specifically mediating Cap2- and ΔCTG-sensitivity to anti-T4 defense. The authors then performed extensive studies to determine and confirm Vs.4’s specific anti-CBASS function: isothermic calorimetry demonstrated Vs.4-cGAMP binding; crystallography revealed Vs.4-hexamer formation with 3 bound cGAMP molecules (Fig. 1) and informed the generation of new mutants at the cGAMP-Vs.4 binding interface to confirm Vs.4 function *in cellulo*, and recombinant Vs.4 and CapV confirmed Vs.4’s function as a CN-sponge in vitro. Furthermore, 198 Vs.4 homologs could be identified in other phages, revealing a whole new class of anti-CBASS proteins (Acb), in addition to the previously reported anti-CBASS, CN-nuclease Acb1. Indeed, a further study confirming these findings,^5^ terms Vs.4 and homologs Acb2 to underscore the significance of this discovery.

Altogether, Jenson et al. breaks important ground in basic research and thus raises fundamental, new questions for future studies: Does Cap2 target other possible ULPs? What other targets might bacterial cGAS conjugate and to what end? Do other proteins without the typical structural hallmarks of ULPs engage in ubiquitin-like conjugation? If so, which ones, how can we possibly identify them and their function? Moreover, novel insight into phage anti-CBASS signaling is not only relevant to the phage-bacteria arms race but also to our own with antibiotic resistance, for which phage therapy is increasingly seen as an important alternative approach. Here, Vs.4, and potentially further Acb-families, may yet have an important role in the clinical treatment of bacterial disease.

## References

[1] Jenson, J. M., Li, T., Du, F., Ea, C.-K. & Chen, Z. J. Ubiquitin-like conjugation by bacterial cGAS enhances anti-phage defence. *Nature*10.1038/s41586-023-05862-7 (2023). 10.1038/s41586-023-05862-7 PMC10097602 36848932

[2] Millman A, Melamed S, Amitai G, Sorek R (2020). Diversity and classification of cyclic-oligonucleotide-based anti-phage signalling systems. Nat. Microbiol..

[3] Cohen D (2019). Cyclic GMP–AMP signalling protects bacteria against viral infection. Nature.

[4] Ledvina HE (2023). An E1–E2 fusion protein primes antiviral immune signalling in bacteria. Nature.

[5] Huiting E (2023). Bacteriophages inhibit and evade cGAS-like immune function in bacteria. Cell.

